# Research on Biomarkers of Different Growth Periods and Different Drying Processes of *Citrus wilsonii* Tanaka Based on Plant Metabolomics

**DOI:** 10.3389/fpls.2021.700367

**Published:** 2021-07-14

**Authors:** Hui Yan, Zong-Jin Pu, Zhen-Yu Zhang, Gui-Sheng Zhou, Dong-Qian Zou, Sheng Guo, Chao Li, Zhi-Lai Zhan, Jin-Ao Duan

**Affiliations:** ^1^Jiangsu Key Laboratory for High Technology Research of TCM Formulae, Jiangsu Collaborative Innovation Center of Chinese Medicinal Resources Industrialization, National and Local Collaborative Engineering Center of Chinese Medicinal Resources Industrialization and Formulae Innovative Medicine, Nanjing University of Chinese Medicine, Nanjing, China; ^2^Jumpcan Pharmaceutical Group Co., Ltd., Taizhou, China; ^3^State Key Laboratory of Dao-di Herbs Breeding Base, National Resource Center for Chinese Materia Medica, China Academy of Chinese Medical Sciences, Beijing, China

**Keywords:** *Citrus wilsonii* Tanaka, plant metabolomics, different drying methods, different growth periods, biomarker, *Citrus*

## Abstract

Fruit of *Citrus wilsonii* Tanaka called as “Xiang yuan” in Chinese, which means fragrant and round. It was widely used in the pharmaceutical and food industries. This fruit has well-known health benefits such as antioxidant, radical scavenging, and anti-inflammatory. Naringin, deacetylnomilin, citric acid, limonin, and nomilin were the characteristic components of *Citrus wilsonii* Tanaka. Although the fruit of *Citrus wilsonii* Tanaka possessed many applications, there was a lack of research on the growth period and drying process. In this study, plant metabolomics was used to analyze the biomarkers of the growth period, and appearance indicators and metabolites abundance were combined for the analysis of change regularities of the growth period. The representative differential metabolites of naringin, citric acid, and limonin were screened out, and the abundance of these components was relatively highest in the middle of the growth period. Therefore, the fruit of *Citrus wilsonii* Tanaka should be harvested before it turned yellow completely, which could effectively ensure the content of potential active ingredients. In the comparison of different drying methods, citric acid and naringin were considered to be representative differential components, but limonoids were relatively stable and not easily affected by drying methods. Naringin was an index component that could not only be reflected the maturity but also related to different drying methods. Considering its physical and chemical properties and its position, naringin had the potential to be a biomarker of *Citrus wilsonii* Tanaka.

## Introduction

Fruit of *Citrus wilsonii* Tanaka (CWT) was used as a traditional medicine in China (Rao et al., 2021), called as Hsiang yuan (Xiang yuan in Chinese), meaning that fragrant and round. This fruit also had a name called Ichang lemon, but the latest researches confirmed that this naming might be inaccurate. *Citrus* species originated from the southeast foothills of the Himalayas, in a region that included the eastern area of Assam, northern Myanmar, and western Yunnan (Gmitter and Hu, 1990), and then widely distributed all over the world (Wu et al., 2018). There were two classic views on the origin of CWT, one of which was that CWT was a putative hybrid of *Citrus ichangensis* and *Citrus maxima* and the other was that CWT was an indigenous variety related to Yuzu (*Citrus junos*). Recent studies confirmed that CWT was an offspring of Yuzu by molecular markers (Shimizu et al., 2016). Further researches speculated that CWT might be a pummelo × Yuzu hybrid, and pummelo might be the maternal parent (Demarcq et al., 2021).

*Citrus wilsonii* Tanaka was widely grown and used in China to perfume rooms and cabinets due to its pleasant sensory value. In the United States, CWT was often used for lemon pie, and consumers who used CWT prefer it to regular pies. However, CWT was not very delicious, because it had too many seeds and its juice was too acidic. Although the direct consumption experience was not good, the dried CWT still was an important Chinese medicine, it exerted the pharmacological role of regulating “qi” according to the theory of traditional Chinese medicine. As a member of the *Citrus* genus, CWT also presented well-known health benefits including antioxidant, radical scavenging, and anti-inflammatory (Denaro et al., 2020). Flavonoids, alkaloids, coumarins, limonoids, phenol acids, and volatile compounds were the main bioactive compounds in CWT. Naringin, deacetylnomilin, citric acid, limonin, and nomilin were the characteristic components of CWT (Zhao et al., 2015). As we all know, in the process of plant growth, the contents of internal compounds were in dynamic change processes, and these changes would further be reflected in the changes of appearance (Lee et al., 2012). Nowadays, CWT possessed a wide range of applications, but there was a lack of in-depth research, especially on the growth period and drying process.

The dynamic changes of the plant growth process were often analyzed based on the methods of plant metabonomics, which had grown into one of the major approaches for research on the metabolites in plants (Isah, 2019). Most researches were performed by ultraperformance liquid chromatography-quadrupole time-of-flight mass spectrometry (UPLC-Q-TOF/MS), gas chromatography-time-of-flight mass spectrometry (GC-TOF/MS), nuclear magnetic resonance (NMR), etc. Among these, the liquid chromatography-mass spectrometry (LC-MS) was usually employed to analyze the metabolites that were non-volatile, polar, or thermally labile, especially phenolic acids and flavonoids (De Vos et al., 2007). When these analytical results coupled with chemometric, an in-depth analysis of the biomarkers in plants could be obtained.

**Figure 1:**
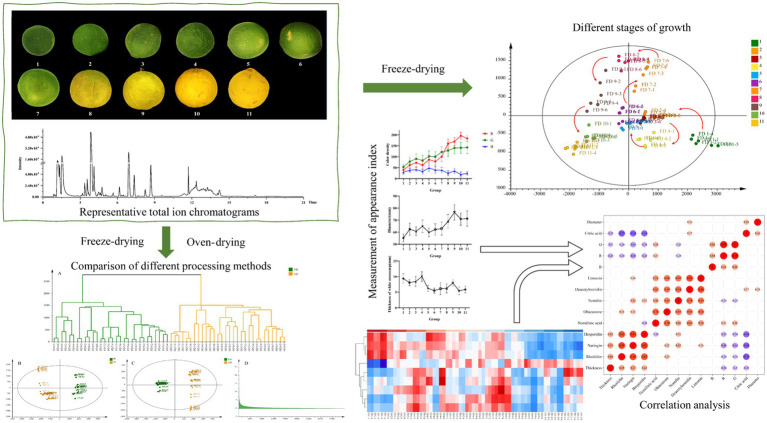
A work-flow for this study.

In this study, some indicators about the appearance of CWT were measured, and then plant metabolomics was used to analyze the biomarkers of the growth period and different drying methods. Heatmap was used to characterize the changing trends of differential metabolites. Appearance indicators and metabolites abundance were combined for correlation analysis to explain the change regularities of the growth period. Figure 1 shows the basic framework of the proposed strategy in this study, which might be employed to profile various metabolites in different sources of CWT samples and would be helpful for finding biomarkers to distinguish the different growth periods and different drying methods of CWT samples in practical applications.

## Materials and Methods

### Chemicals and Reagents

Three chemical standards, including naringin, hesperidin, and limonin, were used in this study and were acquired from Nanjing Liangwei Biotechnology Company (Nanjing, China).

Acetonitrile, methanol, and formic acid (chromatographic grade) were bought from Merck (Darmstadt, Germany). Deionized water was prepared by the Milli-Q water purification system (Millipore, Bedford, MA, United States).

### Samples Preparation

**Figure 2:**
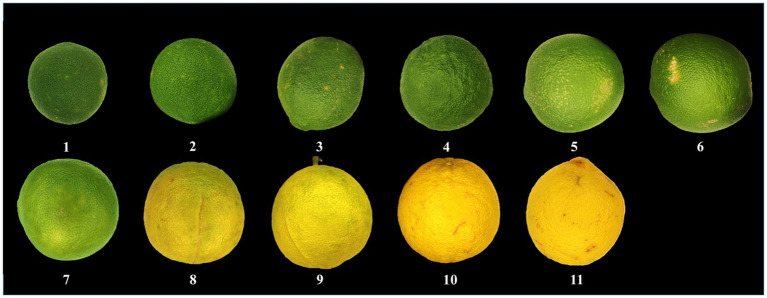
Representative samples of *Citrus wilsonii* Tanaka (CWT).

Eleven groups (groups 1–11) of CWT samples were collected in Dongxing Town (Jingjiang City, Jiangsu Province, China) from July to November 2020, which were identified as CWT by Dr. Hui Yan at the Department of Pharmacy, Nanjing University of Chinese Medicine, Nanjing, China. Each batch included six replicate samples. The collected samples included different ripeness fruits of CWT, and the details of samples are showed in Figure 2.

Each CWT sample was photographed, and each photograph was regularly marked with 20 markers. The values of R(red), G(green), and B(blue) were then extracted from each marker. Meanwhile, we also measured and recorded the diameter of the corresponding CWT sample and the thickness of white mesocarp.

Samples of each batch were divided into two groups for oven-drying (OD) and freeze-drying (FD). Half of the samples were kept in the −80°C ultra-low temperature freezer (Kim and Verpoorte, 2010; Creydt et al., 2018) and then put into a vacuum freeze dryer overnight to remove water. The other half of the samples were processed by oven-drying. All dried samples were pulverized into homogeneous powders (80 mesh) and stored at room temperature under dry conditions. Firstly, 1 g of each sample was weighed accurately into a 50-ml conical flask with a stopper, and then 25 ml of 80% methanol solution added precisely and sealed with parafilm (Perez de Souza et al., 2019). Secondly, all the samples were extracted by ultrasonication for 40 min (40 kHz) at 30°C and then centrifuged at 13,000 rpm/min for 15 min (4°C; Martins Strieder et al., 2019). Thirdly, the supernatants were stored at 4°C and filtered with a 0.22-μm microporous membrane filter before injection.

### UPLC-Q-TOF/MS-Based on Nontarget Analysis

Chromatographic separation was performed on a Waters ACQUITY UPLC system (Waters, Milford, MA, United States). A Waters Acquity UPLC BEH Shield RP C_18_ column (2.1 × 100 mm, 1.7 μm) was used for analysis. The mobile phase included A (0.1% formic acid aqueous solution) and B (acetonitrile; *v*/*v*). The gradient elution procedure was as follows: 0–1 min: 10–36% B; 1–6 min: 36–50% B; 6–8 min: 50% B; 8–9 min: 50–100% B; 9–11 min: 100% B; 11–13 min: 100–50% B; 13–15 min: 50% B; 15–16 min: 50–10% B; and 16–21 min: 10% B. The mobile phase was set at a flow rate of 0.20 ml/min, and the injection volume was 2 μl. The column temperature was set at 30°C, and UV detection wavelength was 200–520 nm.

Mass spectrometry was carried out using a Waters Xevo G2-S QTOF mass spectrometer equipped with an electrospray ionization source (ESI) in negative mode with scan range of *m*/*z* 50–1,200 Da. The mass accuracy and reproducibility were maintained using a LockSpray mode and the [M−H]^−^ ion of leucine–encephalin (*m*/*z* 554.2615 Da) at 100 μl/min (the concentration of 200 pg/ml). The desolvation gas flow rate was set to 800 L/h at desolvation temperature of 400°C. The ion source temperature was held at 120°C. The cone gas flow, extraction cone voltage, capillary voltage, and cone voltage were set to 50 L/h, 4 V, 2.5 kV, and 30 V, respectively. The low collision energy was 6 eV and the high collision energy ranged from 30 to 50 eV.

### Data Processing

Data were pre-processed (peak detection, data mining, alignment, and normalization) by Progenesis QI software (version 4.1; Waters). The processed data were imported to SIMCA-P software (version 14.1; Umetric, Umea, Sweden). Then, principal component analysis (PCA), clustering analysis (CA), partial least square discriminate analysis (PLS-DA), and orthogonal partial least square discriminate analysis (OPLS-DA) were performed by SIMCA-P software. *R*^2^ and *Q*^2^ were employed to investigate the quality and reliability of these multivariate statistical analysis models. Generally, the values of *R*^2^ and *Q*^2^ close to 1.0 indicated an excellent fitness and predictive capability for the model. Variable importance in the projection (VIP) was used to evaluate the importance of each variable in PLS-DA models. The retention time and exact mass of significant compounds were selected and imported back into Progenesis QI for compound identification. The filtered compounds were screened by means of VIP and the value of *p* of ANOVA and were analyzed by Heatmap.

## Results and Discussion

### Measurement of Appearance Index of CWT Samples

**Figure 3:**
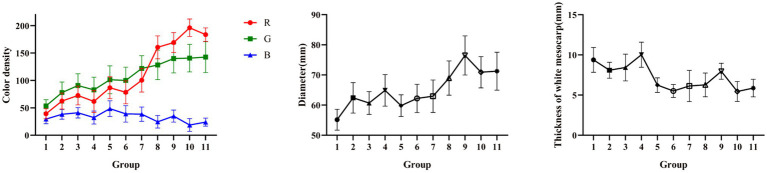
Measurement of appearance index.

In the mature period, the fruit of CWT grown from small to big, from green to yellow, and the results of the study also conformed to this fact. The values of *R* and *G* gradually increased over time, the value of *B* decreased gradually. The constant changes of the above values would eventually lead to the whole phenomenon of changing from green to yellow. The continuous growth of the diameter heralded the process from small to big. The thickness of white mesocarp gradually decreased with increasing maturity. The appearance index mentioned above could reflect the growth status of CWT, and was related to changes in its internal components. The detail appearance index is shown in Figure 3.

### Representative Total Ion Chromatograms of CWT Samples

**Figure 4:**
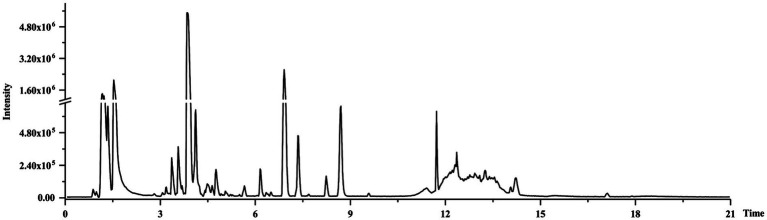
Representative total ion chromatogram.

All samples were tested by UPLC-Q-TOF/MS, total ion chromatograms in the negative mode was shown in Figure 4. Negative ion mode could provide better sensitivity and presented less interference, while positive ion mode could give a better response (Corradini et al., 2011). The visible differences of intensity could be found in the representative total ion chromatograms of samples at different groups. Flavone glycosides were abundant components in CWT, and most flavonoids were detected in the negative ion mode (Justesen, 2000). Also, limonoids could be detected in CWT.

### Cluster Analysis

**Figure 5:**
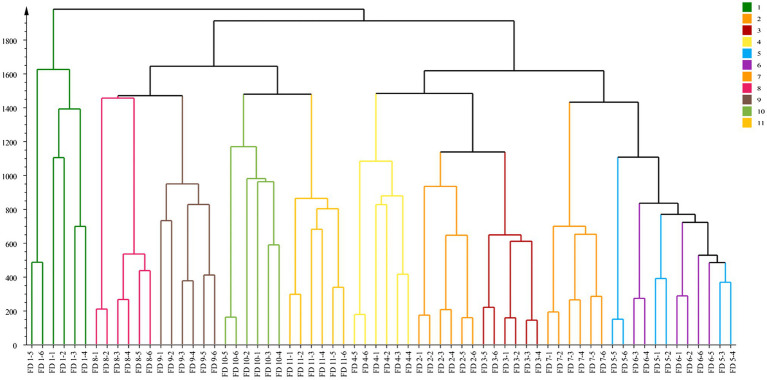
Cluster analysis of CWT at different growth stage in negative ion mode.

The CA based on metabolites is shown in Figure 5. The goal of CA was to classify units so that there was greater similarity between units within groups than between units in different groups (Klastorin, 1983). Results of CA indicated that the samples of each group were preferentially divided into one class, and other groups were different. Groups close to each other indicated similar chemical composition characteristics. This study obtained CA is shown in Figure 5, in which three well-defined clusters were visible. Group 1 was categorized into one cluster; Groups 2–7 were categorized into one cluster while the rest groups were categorized into one cluster. Combined with the appearance of CWT, when the CWT was green and gradually grew up, the similarity between these groups was great. When the CWT gradually turned yellow, these groups had similar secondary metabolites.

### Principal Component Analysis

**Figure 6:**
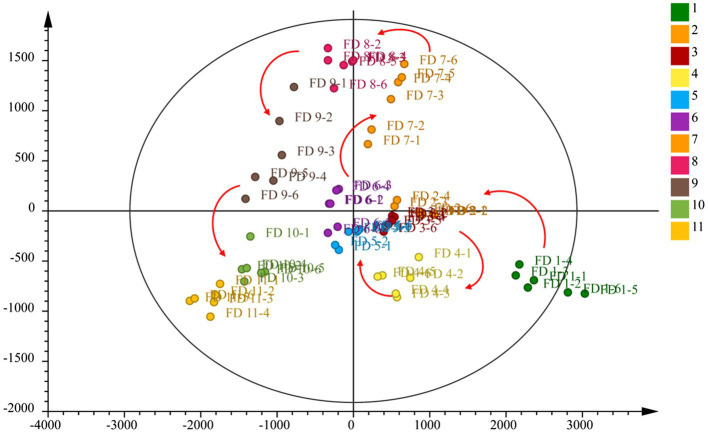
Principal component analysis of CWT at different growth stage in negative ion mode.

To evaluate whether the metabolites profiles could effectively distinguish different growth stage CWT samples, PCA was incipiently selected because it allowed the projection of data from a higher to a lower dimensional space, which was originally used to examine the intrinsic variation in a given data set and provided an overview of the variation among the groups (Zhou et al., 2020). Two model parameters (*R*^2^*X* and *Q*^2^) were often used to assess the quality of PCA. If the values were close to 1, which indicated good fitness and predictive ability of the PCA model (Chang et al., 2017). In this study, the values of *R*^2^*X* (0.946) and *Q*^2^ (0.853), respectively, gave an indication of good fitness and predictability of the PCA model. Samples of the same group could be gathered together, and different groups could be distinguished well. In the score plots of PCA (Figure 6), from group 1 to group 11 had a clear change, corresponding to the growth process of CWT.

### Partial Least Squares Discriminant Analysis and Identification of Metabolites

Compared to PCA, PLS-DA could maximize the separation between groups of observations, hence allowing better classification and prediction capacity (Xie et al., 2008; Liu et al., 2020b). Three model parameters (including *R*^2^*X*, *R*^2^*Y*, and *Q*^2^) were often used to assess the quality of the model. *R*^2^*X* and *R*^2^*Y* represented the explanatory rate of the model to *x* and *y* matrices, respectively. *Q*^2^ showed the predictive ability of the model. The value of *R*^2^*Y* varied from 0 to 1, and 1 indicated a perfect fit. Value of *Q*^2^ > 0.5 indicated good predictive ability, and the value >0.9 represented excellent predictive ability (Malongane et al., 2018). The VIP plot was used to select the most promising relevant variables for classification (Liu et al., 2020a). Value of VIP > 1 indicated the compounds carried the most relevant information for class discrimination (Cho et al., 2008). The loading plots were also used as a reference for the screening of differential metabolites.

**Figure 7:**
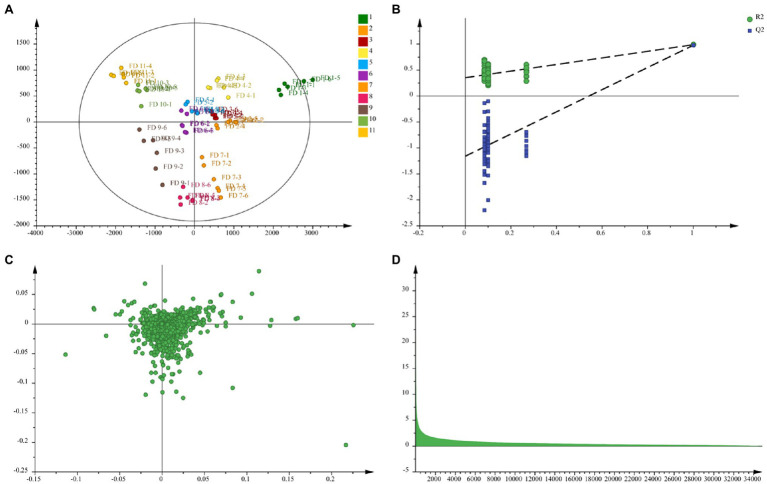
Partial least squares discriminant analysis **(A)**, permutation test **(B)**, loading plot **(C)**, and variable importance in the projection (VIP; **D**) in negative ion mode.

Orthogonal partial least square discriminate analysis was used to achieve the search for differential metabolites in different growth period of CWT samples. For plant metabolomics, PLS-DA score plot revealed the growth period effect on CWT differential metabolites (Figure 7A). The score plot showed clear separation among the 11 experimental groups. Figure 7B shows a good validation of model based on permutation test. The PLS-DA model presented well expiation and prediction ability as reflected by *R*^2^*X* = 0.92, *R*^2^*Y* = 0.962, and *Q*^2^ = 0.885. Finally, nine metabolites were screened and identified as CWT biomarkers associated with growth period based on their loading plot, VIP and values of *p* (Figures 7C,D and Table 1).

**Table 1:** Identification of differential metabolites during the growth period.

No	Retetion time/min	VIP	Compounds	MW	Ion mode	*m*/*z*	MS^2^
1	3.88	24.87	Naringin	580.53	[M−H]^−^	579.19	579, 505, 477, 459, 271
2	6.93	21.15	Deacetylnomilin	472.53	[M+HCOO]^−^	517.22	471, 453, 429, 333
3	1.53	13.42	Citric acid	192.13	[M−H]^−^	191.02	191, 173, 147, 129
4	7.38	11.57	Limonin	470.53	[M+HCOO]^−^	515.21	515, 469, 453, 174
5	3.64	8.20	Rhoifolin	578.53	[M−H]^−^	577.17	565, 427, 313, 269
6	7.31	6.10	Nomilinic acid	532.23	[M−H]^−^	531.24	513, 469, 445, 145
7	8.24	5.82	Nomilin	514.22	[M−H]^−^	513.27	499, 453, 367, 339
8	4.48	3.29	Hesperidin	610.56	[M−H]^−^	609.20	593, 563, 301, 286, 258
9	9.43	1.31	Obacunone	454.51	[M+HCOO]^−^	499.21	499, 453, 437, 367, 311, 174

The precise relative molecular mass of each differential metabolite was determined by the primary mass spectrometry, and the fragmentation information was obtained by the secondary mass spectrometry, referring reported literatures and database, such as HMDB^1^ and METLIN.^2^ Nine differential components were tentatively and/or unequivocally identified (Tian and Schwartz, 2003) in different growth periods CWT samples, and these differential components with significant changes, which were considered to have the potential to be biomarkers for distinguishing growth stage of CWT. Among these differential metabolites, naringin presented the highest value of VIP and significant change in different growth periods CWT samples. Naringin (4',5,7-trihydroxyflavanone 7-rhamnoglucoside), the predominant flavanone obtained from CWT and related *Citrus* species (Zhao et al., 2015), showed many potential biological activities, including anti-inflammatory, antioxidant, antimicrobial, and anticancer (Bacanli et al., 2015). After oral administration, naringin was hydrolyzed to naringenin, which was the main absorbable metabolite. Many previous studies found that naringin and naringenin showed potential anti-coronavirus and anti-inflammatory activity and were promising in the use of prevention and treatment of 2019 novel coronavirus (COVID-19) infection (Tutunchi et al., 2020; Alzaabi et al., 2021). Additionally, a previous report found that the flavonoid-rich extract of CWT could attenuate p-p38 MAPK level and presented potential use for preventing cytokine storm of COVID-19 (Cheng et al., 2020). Therefore, a regimen of CWT as a flavonoid-rich plant could be recommended to supplement a sufficient amount of flavonoids for the protection and treatment from COVID-19 infection.

### Heatmap of Differential Metabolites in Different Groups

**Figure 8:**
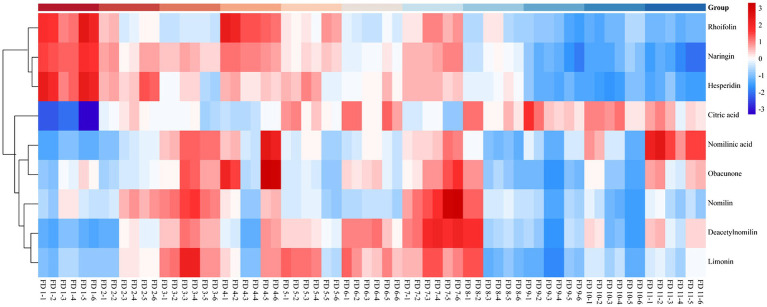
Heatmap of relative content of differential metabolites in different color groups.

Heatmap was an intuitive visualization method for analyzing the distribution of experimental data (Marlin et al., 2019) and could be used for the visualization display of difference data (Xu et al., 2019). Cluster analysis on data could be performed to observe the quality of samples. Each grid represented each metabolite (Huang et al., 2019), and the shade of the color represented the relative content of the metabolite (Theriot et al., 2014). In the tree of cluster analysis, the larger the relative content of each metabolite presented the darker the color (red was upregulated, and blue was downregulated). Each row represented the relative content of each metabolite in different samples, and each column represented the relative content of all metabolites in each sample. The upper tree of cluster analysis was obtained from the result of different metabolites from different samples. The scale of heatmap ranged from −3 to +3. The relative contents of rhoifolin, naringin, and hesperidin gradually decreased during the ripening process of CWT. But after group 8, it showed a significant decrease. Citric acid increased gradually over time. Nomilin, obacunone, deacetynomilin, and limonin were more abundant in the middle of the growth period, and these characteristic limonoids in *Citrus* had similar patterns. Similarly, the relative contents of limonoids were significantly lower than the previous groups after group 8. These results seemed to remind us that the contents of characteristic components were at relatively high levels in the middle stage of CWT’s growth. The detailed results are shown in Figure 8.

### Correlation Analysis of Differential Metabolites and Appearance Index

**Figure 9:**
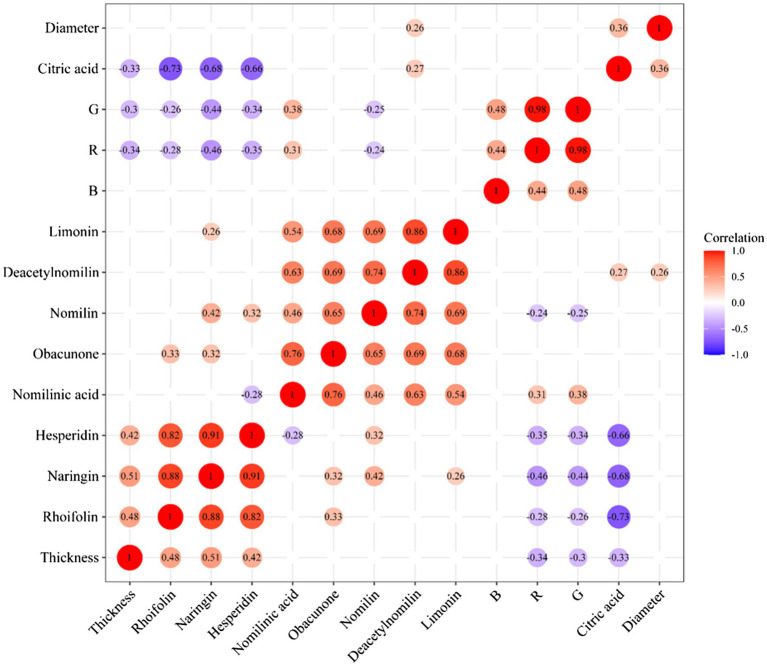
Correlation analysis of differential metabolites and appearance index.

The correlation analysis of heatmap was a graph that analyzed the correlation between two or more variables. Red indicated a positive correlation between two variables, and blue indicated a negative correlation between two variables. The number in each cell indicated a correlation coefficient. A strong positive correlation existed between rhoifolin, naringin, and hesperidin. Additionally, the same result could be found from these limonoids compounds. A strong negative correlation existed between citric acid and flavonoids. There was no significant correlation between rhoifolin, naringin, hesperidin, and thickness of white mesocarp, but the contents of the above three flavonoids and the diameter of CWT presented negative correlation. It was found that these compounds (including nomilin, deacetynomilin, and limonin) and the thickness of the white mesocarp had a positive correlation. In the process of maturity, the thickness of white mesocarp decreased gradually, the contents of limonoids decreased too. There was a correlation between color index (*R*, *G*, and *B*) and internal chemical compounds, but the values of correlation were diminutively ranged from −0.46 to 0.48. These external color indexes could be used as a part of the reference for judging the content of the internal components of CWT. Figure 9 shows the heatmap of correlation analysis of between differential metabolites and appearance index in CWT.

### Effects of Different Drying Methods on CWT

**Figure 10:**
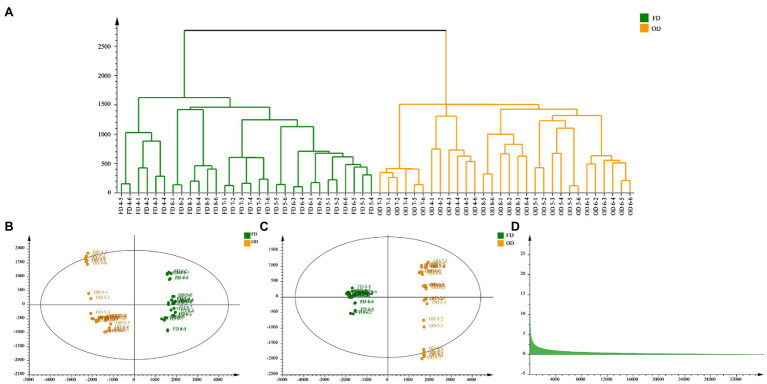
Effects of different drying methods on CWT (**A**: cluster analysis, **B**: principal component analysis, **C**: orthogonal partial least squares discriminant analysis, **D**: VIP).

In China, CWT was usually harvested as Chinese medicinal materials when it turned from green to yellow, that was from group 4 to group 8. The current commonly processing method was oven-drying. Hence, OD and FD were compared to explain the effects of different drying methods based on the samples of group 4, 5, 6, 7, and 8 in this study. Results of CA showed samples of FD and OD could be divided well, which indicated CWT samples processed by different drying methods and had significant differences. The PCA reflected the same results as cluster analysis, and the model parameters were as follows: *R*^2^*X* = 0.956 and *Q*^2^ = 0.889. The OPLS-DA was carried out to reveal the compounds that showed significant changes between different drying methods (Boccard and Rutledge, 2013). Results are shown in the Figure 10. The values of *R*^2^*X*, *R*^2^*Y*, and *Q*^2^ = 0.994 of OPLS-DA model were 0.742, 0.995, and 0.994, respectively. Seven chemical components were initially identified, but the values of VIP were not the same as the previous results. Citric acid became the differential metabolite with the highest VIP value, which might be due to its thermal instability. Naringin also had a high value of VIP, which indicated this component could not only reflect the maturity of CWT but also could be used to judge different drying methods. However, different drying methods had a few effects on limonoids (Table 2).

**Table 2:** Differential metabolites identification of different drying methods.

No	Retetion time/min	VIP	Compounds	MW	Ion mode	*m*/*z*	MS^2^
1	1.53	18.06	Citric acid	192.13	[M−H]^−^	191.02	191, 173, 147, 129
2	3.88	17.83	Naringin	580.53	[M−H]^−^	579.19	579, 505, 477, 459, 271
3	3.64	8.58	Rhoifolin	578.53	[M−H]^−^	577.17	565, 427, 313, 269
4	6.93	5.33	Deacetylnomilin	472.53	[M+HCOO]^−^	517.22	471, 453, 429, 333
5	7.31	3.26	Nomilinic acid	532.23	[M−H]^−^	531.24	513, 469, 445, 145
6	7.38	3.02	Limonin	470.53	[M+HCOO]^−^	515.21	515, 469, 453, 174
7	4.48	2.36	Hesperidin	610.56	[M−H]^−^	609.20	593, 563, 301, 286, 258

*Citrus* was widely grown in subtropical and tropical areas in more than 140 countries and regions (Rao et al., 2021). This wide spread of *Citrus* crop was certainly due to the wide application of *Citrus* in food, cosmetics, and pharmaceutical industry. Nowadays, cultivated *Citrus* was selection, or hybridization of, wild ancestor species whose identity and contribution to *Citrus* domestication remained controversial (Wu et al., 2018). CWT was an important *Citrus* by-product exploited by both pharmaceutical and food industries. However, how CWT trees were domesticated and what pathways were used to synthesize the biomarker naringin in CWT were still largely unknown. Therefore, the further study should focus on the genomes of wild and cultivated CWT using genomic, phylogenetic, and biogeographic analyses and investigate the targeted gene expression analysis for naringin pathway analysis in CWT using molecular biology technology.

## Conclusion

During the maturation process of CWT’s fruits, naringin, deacetylnomilin, citric acid, limonin, rhoifolin, nomilinic acid, nomilin, hesperidin, and obacunone were screened out as differential metabolites. Naringin (the most abundant ingredient) had a significant decrease after group 8 (outer skin turned yellow). Limonoids, such as limonin and nomilin, were abundant in the middle of the growth period, and the relative contents of limonoids were significantly lower than the previous groups after group 8. The contents of flavonoid glycosides decreased as the diameter increased, but limonoids and thickness of white mesocarp had a positive correlation. These color indexes (*R*, *G*, and *B*) also had a positive correlation with internal chemical compounds. All these results seemed to indicate that the best harvest time of CWT was the middle period of growth with high levels of active ingredients. Citric acid and naringin were the differential metabolites with the high VIP values, which reflected their relative content could be affected by different drying methods. However, limonoids were relatively stable and not easily affected by drying methods. Naringin was an index component that could not only be reflected the maturity of CWT but also could be used to judge different drying methods. Considering its physical and chemical properties and its position in CWT, naringin had the potential to be a biomarker of CWT.

## Data Availability Statement

The original contributions presented in the study are included in the article/supplementary material, further inquiries can be directed to the corresponding authors.

## Author Contributions

HY, Z-JP, Z-YZ, G-SZ, D-QZ, and J-AD organized the database. HY, Z-JP, Z-YZ, G-SZ, SG, and CL carried out the image analysis. HY, Z-JP, G-SZ, Z-LZ and J-AD carried out the statistical analysis. HY and Z-JP wrote the first draft of the manuscript. All authors contributed to manuscript revision, conception, and design of the study and read and approved the submitted version.

### Conflict of Interest

CL was employed by Jumpcan Pharmaceutical Group Co., Ltd.

The remaining authors declare that the research was conducted in the absence of any commercial or financial relationships that could be construed as a potential conflict of interest.

## Footnotes

^1^http://www.hmdb.ca

^2^http://metlin.Scripps.edu
